# Tualang Honey Reduced Neuroinflammation and Caspase-3 Activity in Rat Brain after Kainic Acid-Induced Status Epilepticus

**DOI:** 10.1155/2018/7287820

**Published:** 2018-07-15

**Authors:** Nur Shafika Mohd Sairazi, Kuttulebbai N. S. Sirajudeen, Mustapha Muzaimi, Swamy Mummedy, Mohd Asnizam Asari, Siti Amrah Sulaiman

**Affiliations:** ^1^Department of Chemical Pathology, School of Medical Sciences, Health Campus, Universiti Sains Malaysia, 16150 Kubang Kerian, Kota Bharu, Kelantan, Malaysia; ^2^Department of Neurosciences, School of Medical Sciences, Health Campus, Universiti Sains Malaysia, 16150 Kubang Kerian, Kota Bharu, Kelantan, Malaysia; ^3^Department of Anatomy, School of Medical Sciences, Health Campus, Universiti Sains Malaysia, 16150 Kubang Kerian, Kota Bharu, Kelantan, Malaysia; ^4^Department of Pharmacology, School of Medical Sciences, Health Campus, Universiti Sains Malaysia, 16150 Kubang Kerian, Kota Bharu, Kelantan, Malaysia

## Abstract

The protective effect of tualang honey (TH) on neuroinflammation and caspase-3 activity in rat cerebral cortex, cerebellum, and brainstem after kainic acid- (KA-) induced status epilepticus was investigated. Male Sprague-Dawley rats were pretreated orally with TH (1.0 g/kg body weight) five times at 12 h intervals. KA (15 mg/kg body weight) was injected subcutaneously 30 min after last oral treatment. Rats were sacrificed at 2 h, 24 h, and 48 h after KA administration. Neuroinflammation markers and caspase-3 activity were analyzed in different brain regions 2 h, 24 h, and 48 h after KA administration. Administration of KA induced epileptic seizures. KA caused significant (*p < 0.05*) increase in the level of tumor necrosis factor alpha (TNF-*α*), interleukin 1 beta (IL-1*β*), glial fibrillary acidic protein (GFAP), allograft inflammatory factor 1 (AIF-1), and cyclooxygenase-2 (COX-2) and increase in the caspase-3 activity in the rat cerebral cortex, cerebellum, and brainstem at multiple time points. Pretreatment with TH significantly (*p < 0.05*) reduced the elevation of TNF-*α*, IL-1*β*, GFAP, AIF-1, and COX-2 level in those brain regions at multiple time points and attenuated the increased caspase-3 activity in the cerebral cortex. In conclusion, TH reduced neuroinflammation and caspase-3 activity after kainic acid- (KA-) induced status epilepticus.

## 1. Introduction

Excitotoxicity is the major mechanism of neuronal death [1]. Excitotoxicity is a process triggered by the excessive activation of ionotropic glutamate receptors induced by excessive release of excitatory amino acids, such as glutamate which cause excitotoxic neuronal degeneration and eventually neuronal death. Kainic acid (KA) is a powerful neurotoxic analogue of glutamate and an agonist of kainate subtype of ionotropic glutamate receptors. KA has been extensively used to study the mechanism of excitotoxicity-induced neurodegeneration and to establish the model for epileptic due to the ability of KA to induce neuroinflammation and apoptosis in the brain [2]. Glial activation and neuroinflammation are believed to contribute to the development and progression of acute and chronic neurodegeneration [3–11]. KA-induced excitotoxicity is associated with neuroinflammation characterized by activation of microglia and astrocytes and increased level of proinflammatory cytokines, including tumor necrosis factor alpha (TNF-*α*), interleukin-1 beta (IL-1*β*), and interleukin-6 (IL-6). [12], which influence the outcome of neurodegeneration [5, 13–15].

Excitotoxicity is a global insult to brain tissue, though different parts of the brain are affected to varying degrees [16]. Several studies have reported that excitotoxicity induced by KA has caused neurodegeneration in the hippocampus, basal ganglia, piriform cortex, thalamus, amygdala, and parietal cortex [17–19]. Up to our knowledge, there has been a little information concerning the excitotoxic effect of kainic acid on the cerebral cortex, cerebellum, and brainstem. There was a study that demonstrated that KA caused neuronal death in the rat cerebral cortical neurons [20]. Another study has demonstrated that glutamate-induced excitotoxicity caused neuronal death and increased in TNF-*α* expression in the cerebral cortex [21]. Glutamate-induced excitotoxicity also caused neuronal death, microglial cell activation, and elevation of TNF-*α* protein levels in the rat cerebral cortical neurons [20, 22, 23]. In the cerebellum, there are two types of neurons that play dominant roles: Purkinje cells and granule cells. Cerebellum granule cells are widely used as a cellular model to study the mechanisms of kainate receptors-mediated neuronal cell death and are vulnerable to excitotoxins [24–26]. Purkinje cells are highly susceptible to the pathological condition that involved excitotoxicity induced by glutamate and are selectively vulnerable to ischemia [27, 28]. For the brainstem, several studies have demonstrated the effect of glutamate in brain stem [29–31].

Honey is known to contain substantial antioxidant compounds. In Malaysia, honey has been used as a supplementation and in the traditional medicine among the local community. Tualang honey (TH) has been reported to have the highest phenolic compound and flavonoid contents among other Malaysian honeys [32–36]. Many studies reported the beneficial effect of TH including antioxidant effect in diabetic rats [37], protective effect in animal model of menopause [38], and cigarette smoke-induced damage in rat male reproductive system [39], and also in the management of wound-healing [40]. In addition, the neuroprotective effect of TH has been previously reported attenuating the cognitive impairment caused by chronic cerebral hypoperfusion-induced neurodegeneration [41] and ameliorating oxidative stress in the rat midbrain against repeated paraquat exposure [42]. TH supplementation also improved the hippocampus and medial prefrontal cortex morphological impairment in stressed ovariectomized rats [43, 44] and reduced the brain oxidative stress and improved memory performances in rats exposed to noise stress [45, 46]. Our previous study has demonstrated that TH has attenuated oxidative stress in the cerebral cortex, cerebellum, and brain stem of KA-induced excitotoxicity rat [47, 48] and reduced the neurodegeneration induced by KA in the rat cortex [47]. These findings led us to hypothesize that the protective effect of TH could be partly attributed to its antioxidant properties. Therefore, this study was conducted to investigate the potential neuroprotective effect of tualang honey on neuroinflammatory markers [TNF-*α*, Interleukins (IL-1*β*, IL-6, and IL-10), GFAP, AIF-1, 5-Lipoxygenase (5-LOX), and COX-2] and apoptotic marker-caspase-3 activity in the rat cerebral cortex, cerebellum, and brainstem at multiple time points of kainic acid-induced excitotoxicity rat model.

## 2. Materials and Methods

### 2.1. Ethics Approval

The ethical approval was obtained from the Institutional Animal Ethic Committee, Universiti Sains Malaysia (USM) [Approval No.: USM/ Animal Ethics Approval/ 2011/ (68) (305)]. All procedures performed in this study were in accordance with the Institutional Guidelines for the Care and Use of Animals for Scientific Purposes. Eight-week-old male Sprague-Dawley rats (200 - 250 g) were obtained from the Animal Research and Service Center (ARASC), Health Campus USM, Kota Bharu, Kelantan, Malaysia. Animals were individually housed in a well-ventilated room maintained at 21 ± 2°C under a 12-h light/dark cycle. Animals had free access to drinking water and food pellets* ad libitum.* The rats were acclimatized for at least a week and they were observed closely for any abnormality before the experiments started. No abnormalities were observed.

### 2.2. Chemicals

KA was purchased from Sigma-Aldrich Co., St. Louis, Missouri, USA. Topiramate (TPM) was purchased from Tokyo Chemical Industries Co., Ltd., Tokyo, Japan. Diazepam was purchased from Atlantic Laboratories Corp. Ltd., Bangkok, Thailand. All chemicals and reagents were of analytical grade.

### 2.3. Tualang Honey

Tualang honey (AgroMas®) was supplied by the Federal Agricultural Marketing Authority (FAMA), Kedah, Malaysia. The honey was previously filtered, evaporated to 20 % (w/v) water content at 40°C, and then sterilized by gamma irradiation (25 kGy) by SterilGamma (M) Sdn. Bhd., Selangor, Malaysia. The same batch of TH was used throughout the study. The physicochemical characterization of TH has been studied and reported by previous studies [35, 36, 49].

### 2.4. Experimental Design

A total of 72 adult male Sprague-Dawley rats (*N* = 72) weighing between 260 and 320 g (aged 9–11 weeks) were selected and randomly divided into four groups (*n* = 18 rats per major group). Each major group was further randomly divided into three subgroups depending on the time of sacrifice (after 2 h, 24 h, and 48 h of KA administration;* n* = 6 rats per subgroup), with a total of 12 subgroups in each study. The groups are as follows:

1. **Group 1: the saline-treated group (control group)**: rats received drinking water via oral gavage five times every 12 h.
2. **Group 2: the KA only-treated group:** rats received drinking water via oral gavage five times every 12 h.
3. **Group 3: the tualang honey (TH) + KA-treated group:** rats received Tualang honey (1.0 g/kg body weight; diluted in distilled water) via oral gavage five times every 12 h. The dosage of TH at 1.0 g/ body weight was selected based on the previous studies that demonstrated the protective effect of TH in a model of diabetic animals [37] and a model of menopause animals [38] as well as in our previous study [48].
4. **Group 4: the topiramate (TPM) + KA-treated group**: rats received TPM (40 mg/kg body weight; dissolved in 0.9 (w/v) NaCl solution with pH 8.0) via oral gavage five times every 12 h. The topiramate dosage was chosen based on other studies [50–52].

All treatments were conducted between 8:00 a.m. and 10:00 a.m. and between 8:00 p.m. and 10:00 p.m. for each treatment day. Thirty minutes after the last oral administration, animals in all groups received a subcutaneous injection (s.c.) of KA (15 mg/kg body weight; 10 mg/ml in saline). For the control groups, a subcutaneous injection of saline was administered instead.

Considering the fact that the dosage of KA (15 mg/kg body weight) used was associated with high mortality rate, special efforts were made to improve the survival rate of KA-induced animals [48, 53]. Approximately 90 min after the first generalized seizure, which was categorized as stage 4 by study of Zhang et al. [54], diazepam (10 mg/kg body weight) was injected intraperitoneally to animals of 24 h and 48 h subgroup. For the control groups, an equal amount of saline was administered.

### 2.5. Sample Collection and Tissue Homogenate Preparation

The rats were decapitated using guillotine depending on the time of sacrifice: 2 h, 24 h, and 48 h after KA administration with the respective control groups (*n* = 6 rats per groups for each time point). The brain was quickly removed. The cerebral cortex, cerebellum, and brainstem were rapidly dissected out, weighed, and homogenized in an ice-cold sodium phosphate buffer (0.1 M, pH 7.4) to make 7.5 % (w/v) homogenates using a motor-driven Teflon-glass homogenizer. The homogenates were then centrifuged in a refrigerated centrifuge at 1000 x* g *for 15 min at 4°C. Aliquots of the resulting supernatant were used for the determination of biochemical assay.

### 2.6. Measurement of Total Protein

The total protein concentration in each brain region homogenates: cerebral cortex, cerebellum, and brainstem, was determined using a Bio-Rad Quick Start™ Bradford Protein Assay kit (Bio-Rad, USA) based on the principle of Bradford assay [55].

### 2.7. Measurement of Tumor Necrosis Factor Alpha (TNF-*α*) Level

For brain region homogenate, measurement of tumor necrosis factor alpha (TNF-*α*; proinflammatory cytokine) was estimated using commercially available rat TNF-*α* enzyme-linked immunosorbent assay (ELISA) Kit (Wuhan USCN Business Co., Ltd., Wuhan, China) following the manufacturer's protocol. For the serum, quantitative measurement of TNF-*α* was estimated using commercially available rat TNF-*α* platinum ELISA Kit (eBioscience, San Diego, USA) following the manufacturer's protocol.

### 2.8. Measurement of Interleukin 1 Beta (IL-1*β*) Level

For brain region homogenate, measurement of interleukin 1 beta (IL-1*β*; proinflammatory cytokine) was estimated using commercially available rat IL-1*β* ELISA Kit (Wuhan USCN Business Co., Ltd., Wuhan, China) following the manufacturer's protocol. For the serum, quantitative measurement of IL-1*β* was estimated using commercially available rat IL-1*β* platinum ELISA Kit (eBioscience, San Diego, USA) following the manufacturer's protocol.

### 2.9. Measurement of Interleukin 6 (IL-6) Level

Measurement of interleukin 6 (IL-6; proinflammatory cytokine) was estimated using commercially available rat IL-6 ELISA Kit (Wuhan Fine Biological Technology Co., Ltd., China) following the manufacturer's protocol.

### 2.10. Measurement of Interleukin 10 (IL-10) Level

For brain region homogenate, measurement of interleukin 10 (IL-10; anti-inflammatory cytokine) was estimated using commercially available rat IL-10 ELISA Kit (Wuhan USCN Business Co., Ltd., Wuhan, China) following the manufacturer's protocol.

### 2.11. Measurement of Glial Fibrillary Acidic Protein (GFAP) Level

Measurement of glial fibrillary acidic protein (GFAP) level was estimated using commercially available rat glial fibrillary acidic protein ELISA Kit (Wuhan Fine Biotech Co., Ltd., Wuhan, China) following the manufacturer's protocol.

### 2.12. Measurement of Allograft Inflammatory Factor 1 (AIF-1) Level

Measurement of allograft inflammatory factor 1 [AIF-1; also known as ionized calcium-binding adapter molecule 1 (Iba-1)] level was estimated using commercially available rat allograft inflammatory factor 1 ELISA Kit (Shanghai Qayee Biotechnology Co., Ltd., Shanghai, China) following the manufacturer's protocol.

### 2.13. Measurement of Cyclooxygenase-2 (COX-2) Level

Measurement of cyclooxygenase-2 (COX-2) level was estimated using commercially available rat Cyclooxygenase-2 ELISA Kit (Wuhan Fine Biotech Co., Ltd., Wuhan, China) following the manufacturer's protocol.

### 2.14. Measurement of 5-Lipoxygenase (5-LOX) Level

Measurement of 5-lipoxygenase (5-LOX) level was estimated using commercially available rat Arachidonate 5-lipoxygenase ELISA Kit (Wuhan Fine Biotech Co., Ltd., Wuhan, China) following the manufacturer's protocol.

### 2.15. Measurement of Caspase-3 Enzymatic Activity Assay

Caspase-3 enzymatic activity was measured using commercially available rat caspase-3/CPP32 colorimetric assay kit (BioVision, Inc., Milpitas, CA, USA) according to the manufacturer's instruction. The enzymatic reaction for caspase-3 activity was carried out in a 96-well microplate. Each reaction required 50 *μ*l of sample (containing approximately 50 *μ*g of total protein). A 50 *μ*l of 2X Reaction Buffer (containing 10 mM DTT) was added to each sample. Then, a 5 *μ*l of 4 mM caspase-3 substrate, DEVD-pNA (final concentration: 200 *μ*M) was added to each reaction well. The reaction mixtures were mixed well and incubated for 2 h at 37°C. The absorbance was measured using a plate reader at a wavelength of 405 nm. Caspase-3 activity was reported as optical density (OD) measurement at 405 nm [56].

### 2.16. Statistical Analysis

All data were analyzed using IBM Statistical Package for the Social Sciences (SPSS) for Windows software version 22.0 (SPSS Statistics IBM, Chicago, USA). For the normally distributed data with equal variances, the statistical significance of differences was determined by one-way analysis of variance (ANOVA) test followed by Tukey post hoc test. Descriptive statistic results are expressed as Mean ± Standard Error of Mean (SEM). Meanwhile, for the nonnormally distributed data with unequal variances, the statistical significance of differences was determined by Kruskal-Wallis test and followed by Mann–Whitney* U* (MW) test. Descriptive statistic results were expressed as median (interquartile range; IqR). Data were considered significantly different when the* p* value was less than 0.05 (*p*< 0.05).

## 3. Results

### 3.1. KA Administration Induced Epileptic Seizures

KA (15 mg/kg body weight; s.c.) induced epileptic seizures in all KA-treated rats and KA-treated rats that received pretreatment of TH and TPM, in contrast to the control group, which showed no seizure activity.

### 3.2. KA Caused Increase in the Levels of TNF-*α* and IL-1*β* in the Cerebral Cortex, Cerebellum, Brainstem, and Serum and Is Attenuated by Pretreatment of TH and TPM

**Figure 1:**
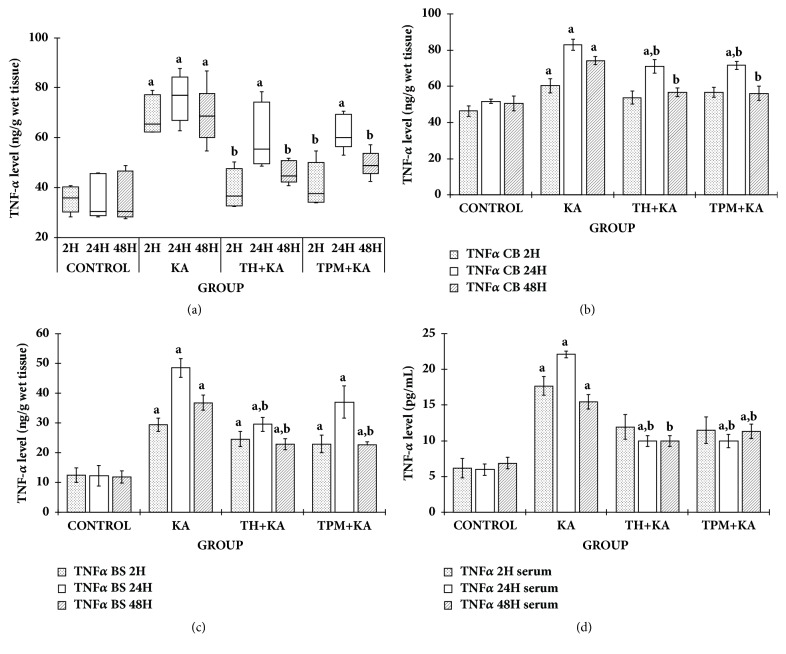
**TNF-** **α** **level in the cerebral cortex, cerebellum, brainstem, and serum of control and kainic acid-induced groups at multiple time points**. Graph showed the levels of TNF-*α* in the cerebral cortex (a), cerebellum (b), brainstem (c), and serum (d) at 2 h, 24 h, and 48 h after KA administration in comparison to the relative control groups. In Figure 1(a), data are presented as median (IqR), n = 6 per group, for each time point. Statistical analysis: Kruskal-Wallis H test followed by Mann–Whitney* U* (MW) test with p<0.05 indicates significant difference. ^a^p<0.05 compared to control group (MW), ^b^p<0.05 compared to KA group (MW). In Figures 1(b), 1(c), and 1(d), data are presented as mean ± SEM, n = 6 per group for each time point. Statistical analysis: one-way ANOVA test followed by Tukey post hoc test with p<0.05 indicates significant difference. ^a^p<0.05 compared to control group, ^b^p<0.05 compared to KA group. ANOVA, one-way analysis of variance; BS, brainstem; CC, cerebral cortex; CB, cerebellum; IqR; interquartile range; KA, kainic acid; TH, tualang honey; TNF-*α*, tumor necrosis factor alpha, TPM, topiramate; SEM, the standard error of the mean.

TNF-*α* and IL-1*β* levels were statistically significantly higher (p<0.05) in the KA only-treated group in the cerebral cortex, cerebellum, brainstem, and serum at 2 h, 24 h, and 48 h after KA administration when compared to the control group. This indicated that KA caused significant increase in the production of proinflammatory cytokines, TNF-*α*, and IL-1*β* (Figure 1 and Table 1).

In the cerebral cortex, pretreatment with TH significantly (p<0.05) attenuated the increase of TNF-*α* at 2 h and 48 h after KA administration and the increase of IL-1*β* levels at 2 h, 24 h, and 48 h after KA administration. Meanwhile, pretreatment with TPM significantly (p<0.05) attenuated the increase of TNF-*α* at 2 h and 48 h after KA administration and the increase of IL-1*β* levels at 2 h, 24 h, and 48 h after KA administration (Figure 1(a) and Table 1).

In the cerebellum, pretreatment with TH significantly (p<0.05) attenuated the increase of TNF-*α* and IL-1*β* levels at 24 h and 48 h after KA administration. Meanwhile, pretreatment with TPM significantly (p<0.05) attenuated the increase of TNF-*α* level at 24 h and 48 h after KA administration and the increase of IL-1*β* level at 2 h, 24 h, and 48 h after KA administration (Figure 1(b) and Table 1).

In the brain stem, pretreatment with TH significantly (p<0.05) attenuated the increase of TNF-*α* and IL-1*β* levels at 24 h and 48 h after KA administration. Meanwhile, pretreatment with TPM significantly (p<0.05) attenuated the increase of TNF-*α* level only at 48 h after KA administration and the increase of IL-1*β* level at 24 h and 48 h after KA administration (Figure 1(c) and Table 1).

In the serum, pretreatment with TH significantly (p<0.05) attenuated the increase of TNF-*α* level at 24 h and 48 h after KA administration and the increase of IL-1*β* level at 2 h and 48 h after KA administration. Meanwhile, pretreatment with TPM significantly (p<0.05) attenuated the increase of TNF-*α* level at 24 h and 48 h after KA administration and the increase of IL-1*β* level only at 48 h after KA administration (Figure 1(d) and Table 1).

### 3.3. No Changes in the Levels of IL-6 and IL-10 in the Cerebral Cortex, Cerebellum, and Brainstem of KA-Induced Rats

**Figure 2:**
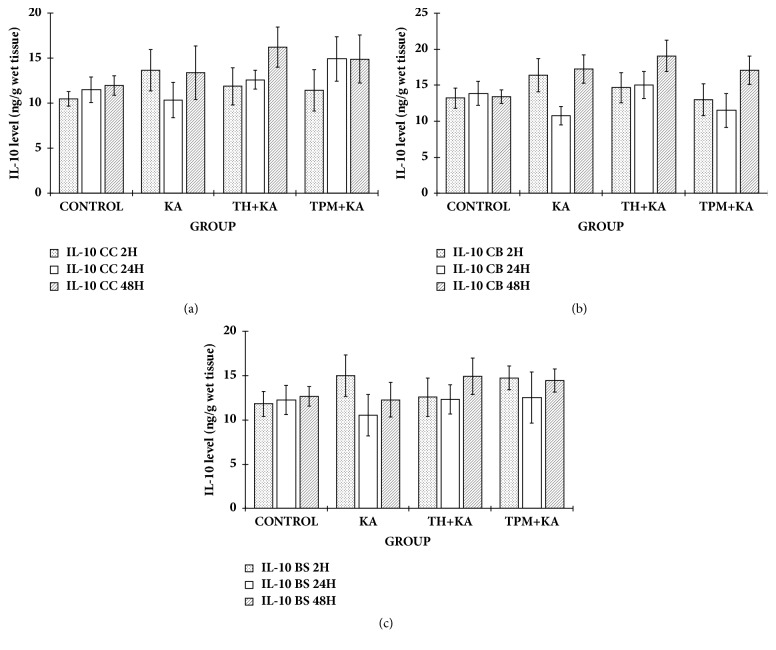
**IL-10 level in the cerebral cortex, cerebellum, and brainstem of control and kainic acid-induced groups at multiple time points**. Graph showed the levels of IL-10 in the cerebral cortex (a), cerebellum (b), and brainstem (c) at 2 h, 24 h, and 48 h after KA administration. Data are presented as Mean ± SEM, n = 6 per group for each time point. Statistical analysis: one-way ANOVA test with p<0.05 indicates significant difference. However, there was no significant difference (p>0.05) in the IL-10 level in all the brain regions or in the rat cerebral cortex, cerebellum, and brainstem among the experimental groups at 2h, 24 h, and 48 h after KA administration. Tukey post hoc test was not performed. ANOVA, one-way analysis of variance; BS, brainstem; CC, cerebral cortex; CB, cerebellum; KA, kainic acid; TH, tualang honey; IL-10, interleukin-10; TPM, topiramate; SEM, the standard error of the mean.

**Figure 3:**
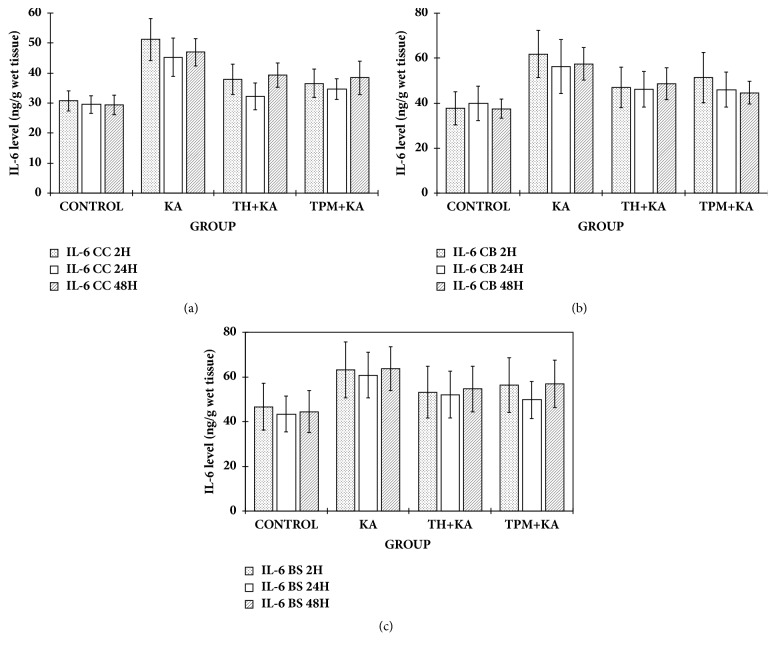
**IL-6 level in the cerebral cortex, cerebellum, and brainstem of control and kainic acid-induced groups at multiple time points**. Graph showed the levels of IL-6 in the cerebral cortex (a), cerebellum (b), and brainstem (c) at 2 h, 24 h, and 48 h after KA administration. Data are presented as Mean ± SEM, n = 6 per group for each time point. Statistical analysis: one-way ANOVA test with p<0.05 indicates significant difference. However, there was no significant difference (p>0.05) in the IL-6 level in all brain regions or in the rat cerebral cortex, cerebellum, and brainstem among the experimental groups at 2h, 24 h, and 48 h after KA administration. Tukey post hoc test was not performed. ANOVA, one-way analysis of variance; BS, brainstem; CC, cerebral cortex; CB, cerebellum; KA, kainic acid; TH, tualang honey; IL-6, interleukin-6; TPM, topiramate; SEM, the standard error of the mean.

There were no significant differences (p>0.05) in the levels of IL-6 and IL-10 in the cerebral cortex, cerebellum, and brainstem among experimental groups at 2 h, 24 h, and 48 h after KA administration (Figures 2 and 3).

### 3.4. KA Caused Increase in the GFAP Level in the Cerebral Cortex, Cerebellum, and Brainstem and Is Attenuated by Pretreatment of TH and TPM

GFAP level was statistically significantly higher (p<0.05) in the KA only-treated group at 2 h and 24 h after KA administration in the cerebral cortex and at 2 h, 24 h, and 48 h in the cerebellum and brainstem when compared to the control group (Table 2). In the cerebral cortex, pretreatment with TH and TPM did not show any significant effect on the increase of GFAP level at 2 h, 24 h, and 48 h after KA administration (Table 2). In the cerebellum, pretreatment with TH significantly (p<0.05) attenuated the increase of GFAP level at 2 h and 48 h after KA administration. Meanwhile, pretreatment with TPM did not show any significant effect on the increase of GFAP level in the cerebellum at 2 h, 24 h, and 48 h after KA administration (Table 2). In the brain stem, pretreatment with TH and TPM did not show any significant effect on the increase of GFAP level at 2 h, 24 h, and 48 h after KA administration (Table 2).

### 3.5. KA Caused Increase in the AIF-1 Protein Level in the Cerebral Cortex and Cerebellum, but Not in the Brain Stem, and Is Attenuated by Pretreatment of TH and TPM

**Figure 4:**
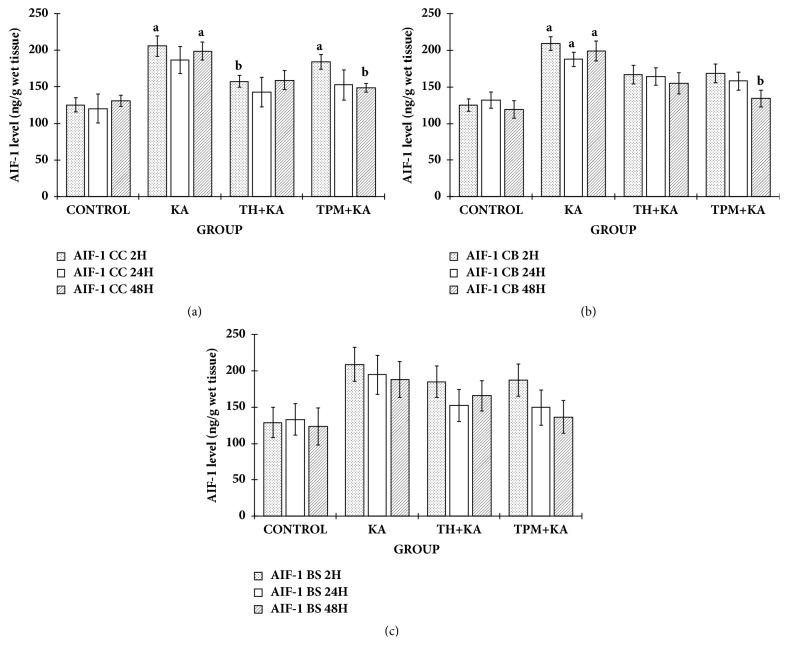
**AIF-1 level in the cerebral cortex, cerebellum, and brainstem of control and KA-induced groups at multiple time points**. Graph showed the levels of AIF-1 in the cerebral cortex (a), cerebellum (b), and brainstem (c) at 2 h, 24 h, and 48 h after KA administration in comparison to the relative control groups. Data are presented as Mean ± SEM, n = 6 per group for each time point. Statistical analysis: one-way ANOVA test followed by Tukey post hoc test with p<0.05 indicates significant difference. ^a^p<0.05 compared to control group, ^b^p<0.05 compared to KA group. AIF-1, allograft inflammatory factor 1; ANOVA, one-way analysis of variance; BS, brainstem; CC, cerebral cortex; CB, cerebellum; KA, kainic acid; TH, Tualang honey; TPM, topiramate; SEM, the standard error of the mean.

AIF-1 level was statistically significantly higher (p<0.05) in the KA only-treated group at 2 h and 48 h in the cerebral cortex and at 2 h, 24 h, and 48 h in the cerebellum when compared to the control group (Figure 4). However, there was no significant difference (p>0.05) in the AIF level in the brainstem among experimental groups at 2 h, 24 h, and 48 h after KA administration (Figure 4). Pretreatment with TH significantly (p<0.05) attenuated the increase of AIF-1 level in the cerebral cortex at 2 h after KA administration (Figure 4). Meanwhile, pretreatment with TPM significantly (p<0.05) attenuated the increase of AIF-1 level in the cerebral cortex and cerebellum at 48 h after KA administration (Figure 4).

### 3.6. KA Caused Increase in the COX-2 Level in the Cerebral Cortex, Cerebellum, and Brainstem and Is Attenuated by Pretreatment of TH and TPM

**Figure 5:**
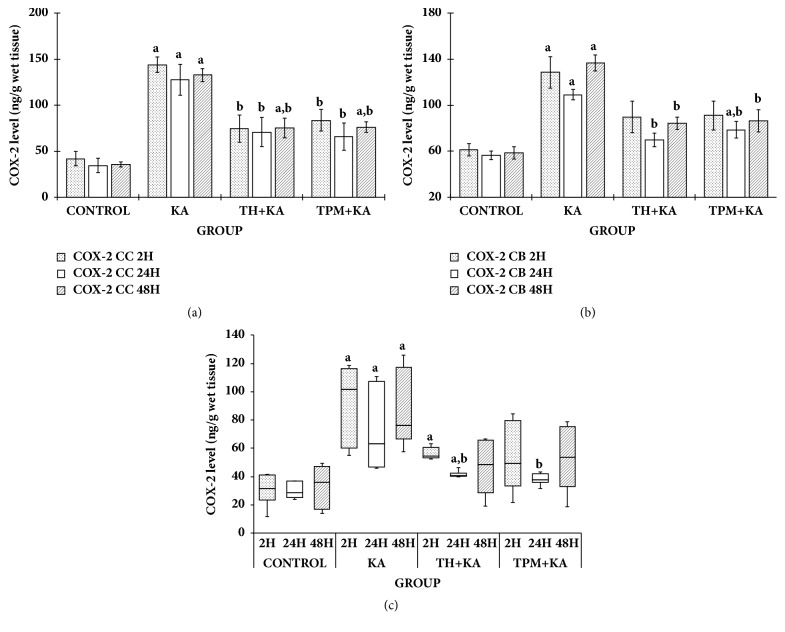
**COX-2 level in the cerebral cortex, cerebellum, and brainstem of control and KA-induced groups at multiple time points**. Graph showed the levels of COX-2 in the cerebral cortex (a), cerebellum (b), and brainstem (c) at 2 h, 24 h, and 48 h after KA administration in comparison to the relative control groups. In Figures 1(a) and 1(b), data are presented as mean ± SEM, n = 6 per group for each time point. Statistical analysis: one-way ANOVA test followed by Tukey post hoc test with p<0.05 indicates significant difference. ^a^p<0.05 compared to control group, ^b^p<0.05 compared to KA group. In Figure 1(c), data are presented as median (IqR), n = 6 per group for each time point. Statistical analysis: Kruskal-Wallis H test followed by Mann–Whitney* U* (MW) test with p<0.05 indicating significant difference. ^a^p<0.05 compared to control group (MW), ^b^p<0.05 compared to KA group (MW). ANOVA, one-way analysis of variance; BS, brainstem; CC, cerebral cortex; CB, cerebellum; COX-2, cyclooxygenase-2; IqR, interquartile range; KA, kainic acid; TH, Tualang honey; TPM, topiramate; SEM, the standard error of the mean.

COX-2 level was statistically significantly higher (p<0.05) in the KA only-treated group in the cerebral cortex, cerebellum, and brainstem at 2 h, 24 h, and 48 h after KA administration when compared to the control group (Figure 5). In the cerebral cortex, pretreatment with TH and TPM significantly (p<0.05) attenuated the increase of COX-2 level at 2 h, 24 h, and 48 h after KA administration (Figure 5(a)). In the cerebellum, pretreatment with TH and TPM significantly (p<0.05) attenuated the increase of COX-2 level at 24 h and 48 h after KA administration (Figure 5(b)). In the brain stem, pretreatment with TH and TPM significantly (p<0.05) attenuated the increase of COX-2 level only at 24 h after KA administration (Figure 5(c)).

### 3.7. KA Caused Increase in 5-LOX Level in the Cerebral Cortex and Cerebellum, but Not in the Brain Stem, and Is Attenuated by Pretreatment of TH and TPM

**Figure 6:**
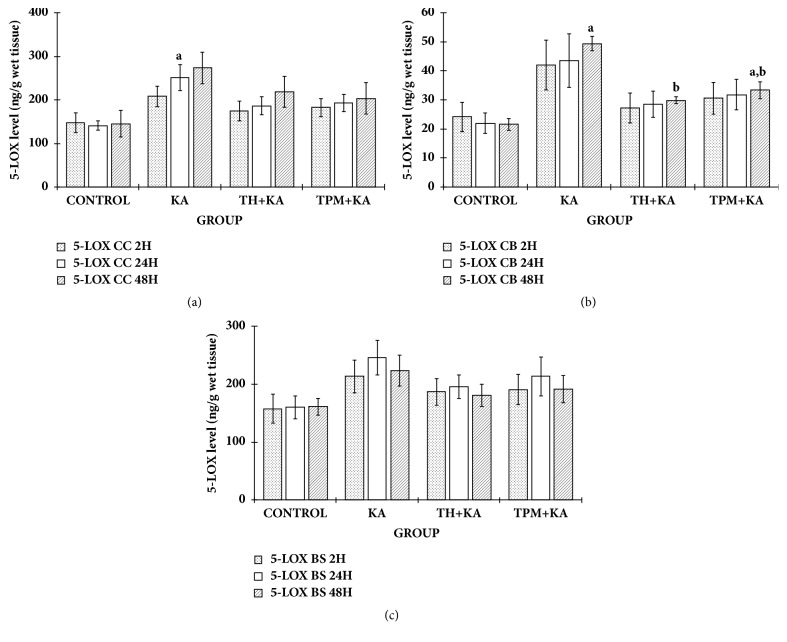
**5-LOX level in the cerebral cortex, cerebellum, and brainstem of control and KA-induced groups at multiple time points**. Graph showed the levels of 5-LOX in the cerebral cortex (a), cerebellum (b), and brainstem (c) at 2 h, 24 h, and 48 h after KA administration in comparison to the relative control groups. Data are presented as Mean ± SEM, n = 6 per group for each time point. Statistical analysis: one-way ANOVA test followed by Tukey post hoc test with p<0.05 indicating significant difference. ^a^p<0.05 compared to control group, ^b^p<0.05 compared to KA group. ANOVA, one-way analysis of variance; BS, brainstem; CC, cerebral cortex; CB, cerebellum; KA, kainic acid; 5-LOX, 5-lipoxygenase; TH, tualang honey; TPM, topiramate; SEM, the standard error of the mean.

5-LOX level was statistically significantly higher (p<0.05) in the KA only-treated group at 24 h after KA administration in the cerebral cortex and at 48 h in the cerebellum when compared to the control group (Figures 6(a) and 6(b)). However, there was no significant difference (p>0.05) in the 5-LOX level in the brainstem among experimental groups at 2 h, 24 h, and 48 h after KA administration (Figure 6(c)). In the cerebral cortex, pretreatment with TH and TPM did not show any significant effect on the increase of 5-LOX level at 24 h after KA administration (Figure 6(a)). In the cerebellum, pretreatment with TH and TPM significantly (p<0.05) attenuated the increase of 5-LOX level at 48 h after KA administration (Figure 6(b)).

### 3.8. KA Caused Increase in the Activity of Caspases-3 in the Cerebral Cortex, Cerebellum, and Brainstem and Is Attenuated by Pretreatment of TH and TPM

**Figure 7:**
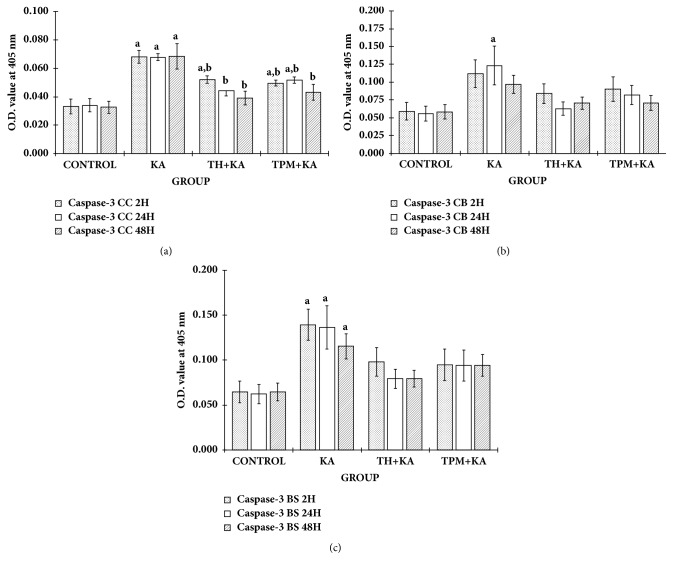
**Caspase-3 activity in the cerebral cortex, cerebellum, and brainstem of control and KA-induced groups at multiple time points**. Graph showed the activity of caspase-3 in the cerebral cortex (a), cerebellum (b), and brainstem (c) at 2 h, 24 h, and 48 h after KA administration in comparison to the relative control groups. Data are presented as Mean ± SEM, n = 6 per group for each time point. Statistical analysis: one-way ANOVA test followed by Tukey post hoc test with p<0.05 indicating significant difference. ^a^p<0.05 compared to control group, ^b^p<0.05 compared to KA group. ANOVA, one-way analysis of variance; BS, brainstem; CC, cerebral cortex; CB, cerebellum; KA, kainic acid; OD, optical density; TH, Tualang honey; TPM, topiramate; SEM, the standard error of the mean.

The apoptosis-related marker (caspase-3) was evaluated. The activity of caspases-3 in the cerebral cortex and brain stem was significantly higher (p<0.05) in the KA only-treated group at 2 h, 24 h, and 48 h after KA administration when compared to the control group (Figures 7(a) and 7(c)). However, in the cerebellum, caspases-3 activity was significantly higher (p<0.05) in the KA only-treated group only at 24 h after KA administration when compared to the control group (Figure 7(b)). Pretreatment with TH and TPM significantly (p<0.05) attenuated the increase of caspases-3 activity only in the cerebral cortex at 2 h, 24 h, and 48 h after KA administration (Figure 7(a)).

## 4. Discussion

In the present study, administration of KA (15 mg/kg body weight; s.c.) resulted in epileptic seizures. All KA-treated groups and KA-treated groups that received pretreatment of TH and TPM had a status epilepticus. This is consistent with previous studies using the same dosage of KA which reported that all KA-treated animals have epileptic seizures [7, 57–59]. Administration of KA has also been known to induce a sequence of well-characterized seizures syndrome [54, 60].

Neuroinflammation and apoptosis contribute considerably to the brain damage occurring after acute injury and adversely affect neurological outcome [2, 9, 57, 61]. KA-induced excitotoxicity is associated with the activation of microglia and astrocytes and these inflammatory processes are induced by enhanced reactive oxygen species (ROS) production and production of proinflammatory mediators, including TNF-*α*, IL-1, IL-6, and COX-2 [2, 11, 12, 62–64]. Inflammatory cytokines have been implicated as a mediator as well as a modulator of neurodegeneration and are induced in response to brain injury [65].

The present study showed that KA caused significant increase in levels of TNF-*α* and IL-1*β* in the cerebral cortex, cerebellum, brainstem, and serum at multiple time points. However, there were no changes in the level of IL-6 and IL-10 in the cerebral cortex, cerebellum, and brainstem at all time points. This finding is consistent with previous studies that showed increase in the level of TNF-*α* and IL-1*β* after KA administration in the different brain region and serum [61, 66, 67]. Other studies reported that IL-6 expression is significantly elevated in the parietal cortex, hippocampus, and amygdala of KA rats following KA injection [67, 68]. Concomitantly, KA also caused significant increase in GFAP level in the cerebral cortex, cerebellum, and brainstem and cause increased in AIF-1 level in the cerebral cortex and cerebellum. This indicated the activation of microglial and astrocytes in those brain regions. Pretreatment with TH attenuated microglial and astrocytes activation (reducing the elevation of GFAP and AIF-1 level) and attenuated the proinflammatory cytokine production (reduced the elevation of TNF-*σ* and IL-1*β* levels) concomitantly. Therefore, this finding suggests that TH exerts its neuroprotective effect via its anti-inflammatory mechanism in this KA induced excitotoxicity model.

In this present study, KA caused significant increase in COX-2 level in the cerebral cortex, cerebellum, and brainstem and increase in 5-LOX level in the cerebral cortex and cerebellum and these increases were reduced by pretreatment with TH. Studies have showed that KA rapidly caused marked increases in COX-2 and 5-LOX mRNA and protein levels following kainate injections in different brain region [69, 70]. KA cause the overactivation of glutamate receptors, causing the influx of Ca^2+^ into neurons. The accumulation of Ca^2+^ activates phospholipase A_2_ which accelerates the formation of arachidonic acid [71]. Therefore, it provides arachidonic acid substrate for COX-2 and 5-LOX activity and arachidonic acid is rapidly metabolized by COX-2 and 5-LOX to inflammatory metabolites, prostaglandins, and leukotrienes, respectively. The neurochemical consequences of increased COX-2 and 5-LOX activities and high levels of their metabolism products (prostaglandin and leukotrienes) could induce inflammatory reactions and also generate oxygen free radical with damaging effects on neural membrane phospholipids, proteins, and DNA [71–74]. Findings from Baran et al. [75] and Minutoli et al. [76] studies have suggested that the protection against KA-induced neurodegeneration via combination of COX and LOX inhibitor pathways or via dual inhibitor of COX and LOX pathways was effective in attenuating KA-induced excitotoxicity. The inhibition of COX-2 and 5-LOX could block leukotriene and prostaglandins production. This enhances the synergistic protective effect, directly through the anti-inflammatory mechanisms and indirectly through the antiradical activity. This may well indicate that blocking of both COX and LOX pathways is necessary to protect the brain against KA-induced seizures and KA-induced neurotoxicity. The free radicals are formed from prostaglandins synthesis and studies have demonstrated the formation of free radicals due to the action of KA [77, 78]. Oxidative stress has been suggested to be a major player in the mechanism of excitotoxicity in different brain regions in KA-induced rats [79, 80]. From our previous studies the antioxidative effects of TH were observed; that is, TH attenuated the oxidative stress in the cerebral cortex, cerebellum, and brainstem of KA-induced rats [47, 48]. Subsequently, TH reduced neurodegeneration in the piriform cortex [47]. From these findings, the reduction of COX-2 and 5-LOX level in addition to the attenuation of oxidative stress could be another mechanism explaining the neuroprotection against KA-induced excitotoxicity by TH.

Neuronal apoptosis also plays a role in neuronal death in the neurodegenerative process [81]. Apoptosis is a highly regulated form of cell death characterized by cell shrinkage, chromatin condensation, DNA fragmentation, and membrane cell death. This energy-dependent process requires ATP for protein synthesis and signal activation where ATP threshold is required for a cell to undergo apoptosis [82]. Mitochondria is the site of oxidative phosphorylation and cellular respiration and plays a role in maintaining a low concentration of calcium in the cytosol. Accumulation of intracellular calcium and the generation of ROS cause the collapse of mitochondrial membrane potential and the opening of mitochondrial transition pores [82]. This will lead to the release of mitochondrial protein (cytochrome-c and apoptotic-inducing factor) located in the mitochondrial intermembrane space via mitochondrial transition pores into cytosol [83]. In caspase-dependent mechanism, cytochrome-c binds to apoptotic protease-activating factor-1 and procaspase-9 to form apoptosome complex and the activation of caspase-3 pathway. In caspase-independent mechanism, apoptotic-inducing factor translocates to nucleus and induces DNA fragmentation and chromatin condensation [84]. Caspase-3 is an indicator of neuronal apoptosis and participates in various apoptosis pathways including KA-induced excitotoxicity [67]. Caspase-3 cleaves most of cellular substance in apoptotic cells and is the main downstream effector caspase. In this study, the caspase-3 activity was increased, particularly in the cerebral cortex and brain stem regions, and the activity level was detected as early as 2 hours. This indicated that the neuronal apoptosis occurs at early stage of excitotoxic insult. This finding is consistent with previous studies that showed increasing in the caspase-3 activity after KA administration at different brain region [66, 67]. Pretreatment with TH attenuated the increased of caspase-3 activity in the cerebral cortex at all time points. Study on propolis showed that propolis prevented the caspase-3 induced by KA in the different brain regions. The beneficial action of propolis is attributed to the presence of phenolic compounds and flavonoids via its antiapoptotic property [66]. Therefore, it could be postulated that TH has antiapoptotic property that could contribute to its neuroprotective effect against KA, especially in the cerebral cortex.

TH has been reported to contain many bioactive compounds, which include gallic acid, syringic acid, coumaric acid, cinnamic acid, caffeic acid, catechin, quercetin, pinobanksin-3-O-propionate, pinobanksin-3-O-butyratengenin, and naringenin [36, 49]. Several studies have investigated the protective effects of compounds, which could be found in TH, against KA model of excitotoxicity. Gallic acid is a phenolic acid that is found not only in plants but also in honey. Study has demonstrated that gallic acid decreased Ca^2+^ release in PC12 cells, ROS production and lipid peroxidation, caspase-3 activity, COX-2 expression, and PGE_2_ production in KA-induced PC12 cells [85]. Gallic acid also prevented amyloid beta protein- (25-35-) induced apoptotic death of cortical neurons* in vitro *by inhibiting glutamate release, ROS production, and calcium release [86]. Another important antioxidant, caffeic acid, is a type of phenolic acid that is found in honey. A study in KA-induced excitotoxicity model has shown that caffeic acid treatment significantly attenuated oxidative stress, acetyl cholinesterase activity, and TNF-*α* level and restored the mitochondrial enzyme complex activity [87]. Propolis has been reported to attenuate KA-induced oxidative stress, TNF-*α* level, and caspase-3 activity in the different brain regions which is attributed to the presence of phenolic compounds and flavonoids. [66, 80, 88]. Thus, the protective effect of TH on oxidative stress, neuroinflammation, and apoptosis may be due to the presence of flavonoids and phenolic acids and the synergistic effect between these bioactive compounds could protect brain against KA-induced excitotoxicity.

This study also determined the significance of the modulatory effect on kainite receptors, by comparing pretreatment between TH and TPM to extend our understanding of the pharmacological mechanism of TH. Pretreatment with TH and TPM helped to reduce the neuroinflammation and apoptosis. Previous studies have reported that TPM reduced lipid peroxidation and caspase-3 activity in piriform cortex of KA-induced rat [58] and attenuated KA-induced hippocampal neurodegeneration in mice [89]. This could be due to the inhibition of the GLuR5 kainate receptors by topiramate through a postsynaptic mechanism [90, 91]. Therefore, it can be suggested that the inhibition of the GLuR5 kainate receptors is essential to protect the brain against KA-induced excitotoxicity. Experimental evidences also indicated that TPM acts through multiple mechanisms of action, including modulation of voltage-dependent sodium channels and voltage-dependent calcium channels [92, 93], inhibition of excitatory glutamate pathway [94], and enhancement of gamma-aminobutyric acid (GABA) activity [95]. Taken together, the protection against excitotoxicity is not based on a specific mechanism or pathway but also on several mechanisms or pathways. The neuroprotective effect of TH in an animal model of KA-induced excitotoxicity is the result of multiple mechanisms, and one of these may be associated with the reduction of inflammatory processes and apoptosis. From our current and previous findings [47, 48], TH can significantly reduce (1) inflammatory responses, including production of proinflammatory cytokines and microglial and astrocytes activation; (2) oxidative stress; and (3) apoptosis and progression of neuronal damage. However, further investigations are needed to elucidate the molecular mechanism of action of TH on apoptotic pathways and neuroinflammation in KA-induced excitotoxicity model.

## 5. Conclusion

Pretreatment with TH reduced neuroinflammation and caspase-3 activity after KA-induced status epilepticus in the cerebral cortex, cerebellum, and brainstem at multiple time points. In conclusion, TH has neuroprotective potential and it can be used as a potential therapeutic measure against neuroinflammation and apoptosis via its antioxidant, anti-inflammatory, and antiapoptotic properties.

## Figures and Tables

**Table 1:** IL-1*β* level in the cerebral cortex, cerebellum, brainstem, and serum of control and kainic acid-induced groups at multiple time points.

	Subgroups	IL-1*β* level				Kruskal-Wallis H test/ANOVA test
		(n = 6 per group for each time point)				
		CONTROL	KA	TH + KA	TPM + KA	*p* value
Cerebral Cortex Median (IqR) (ng/g wet tissue)	2 h	4.16 (0.31)	5.98 (0.26)^a^	5.19 (0.83)^a,b^	5.41 (0.82)^a,b^	*<0.001*
	24 h	3.98 (0.35)	10.31 (0.62)^a^	7.20 (0.68)^a,b^	6.81 (0.63)^a,b^	*<0.001*
	48 h	4.24 (0.14)	8.61 (0.52)^a^	6.43 (1.00)^a,b^	6.46 (0.43)^a,b^	*<0.001*

Cerebellum Median (IqR) (ng/g wet tissue)	2 h	6.26 (0.64)	10.06 (0.80)^a^	8.56 (2.41)^a^	8.62 (1.10) ^a,b^	0.001
	24 h	6.44 (0.95)	13.82 (1.15)^a^	10.55 (0.63)^a,b^	11.20 (0.78) ^a,b^	*<0.001*
	48 h	6.38 (0.62)	12.25 (0.64)^a^	9.94 (0.68)^a,b^	10.71 (1.07) ^a,b^	*<0.001*

Brainstem Median (IqR) (ng/g wet tissue)	2 h	5.07 (0.73)	8.71 (1.92)^a^	8.03 (2.10)^a^	7.80 (1.94)^a^	0.002
	24 h	4.94 (0.75)	10.20 (1.32)^a^	7.53 (0.60)^a,b^	7.12 (0.94)^a,b^	*<0.001*
	48 h	5.07 (0.37)	8.35 (0.51)^a^	6.70 (0.87)^a,b^	6.89 (0.52)^a,b^	*<0.001*

Serum Mean ± SEM (pg/mL)	2 h	12.92 ± 1.19	28.75 ± 3.08^a^	20.83 ± 1.79^a,b^	21.25 ± 1.07^a^	*<0.001*
	24 h	14.58 ± 1.98	47.50 ± 3.54^a^	35.00 ± 5.00^a^	38.25 ± 6.37^a^	*<0.001*
	48 h	11.25 ± 1.91	31.25 ± 2.48^a^	18.33 ± 1.05^b^	19.58 ± 2.77^b^	*<0.001*

For the cerebral cortex, cerebellum, and brainstem, descriptive data are presented as the median (IqR**)** (n = 6 rats per group for each time point) and the significant difference was determined by nonparametric test, Kruskal-Wallis test, followed by Mann–Whitney *U* (MW) post hoc test with *p<0.05 *indicating significant difference. ^a^*p < 0.05 *versus control group (MW); ^b^*p < 0.05 *versus KA group (MW). For the serum, descriptive data are presented as the mean ± SEM (n = 6 rats per group for each time point) and the significant difference was determined by parametric test, ANOVA test, followed by Tukey post hoc test with *p<0.05 *indicating significant difference. ^a^*p < 0.05 *versus control group; ^b^*p < 0.05 *versus KA group. ANOVA, one-way analysis of variance; IL-1*β*, interleukin 1 beta; IqR; interquartile range; KA, kainic acid; TH, tualang honey; TPM, topiramate; SEM, the standard error of the mean.

**Table 2:** GFAP level in the cerebral cortex, cerebellum, and brainstem of control and KA-induced groups at multiple time points.

	Subgroups	GFAP level (ng/g wet tissue)				Kruskal-Wallis H test/ANOVA test
		(n = 6 per group for each time point)				
		CONTROL	KA	TH + KA	TPM + KA	*p* value
Cerebral Cortex Median (IqR)	2 h	6.15 (3.57)	17.88 (15.89)^a^	11.40 (2.26)^a^	10.98 (3.03)^a^	0.001
	24 h	5.61 (6.57)	14.17 (15.43)^a^	11.14 (5.23)	11.24 (5.49)	0.019
	48 h	4.45 (5.51)	11.71 (13.32)	8.96 (7.73)	8.44 (10.50)	0.056

Cerebellum Median (IqR)	2 h	3.66 (1.24)	8.85 (5.05)^a^	4.95 (2.01)^b^	5.73 (1.82)^a^	0.001
	24 h	3.74 (1.78)	10.76 (5.99)^a^	4.57 (0.98)	4.95 (3.57)	0.006
	48 h	2.98 (0.46)	7.03 (5.96)^a^	3.80 (1.30)^b^	5.21 (1.96)^a^	0.001

Brainstem Mean ± SEM	2 h	10.19 ± 1.98	21.28 ± 1.90^a^	14.13 ± 1.36	15.71 ± 2.31	0.005
	24 h	9.71 ± 1.14	22.06 ± 3.65^a^	14.11 ± 2.71	15.20 ± 1.87	0.020
	48 h	9.12 ± 0.95	16.86 ± 1.55^a^	12.59 ± 1.42	12.30 ± 1.37	0.006

For the cerebral cortex and cerebellum, descriptive data are presented as the median (IqR**)** (n = 6 rats per group for each time point) and the significant difference was determined by nonparametric test, Kruskal-Wallis test, followed by Mann–Whitney *U* (MW) post hoc test with *p<0.05 *indicating significant difference. ^a^*p < 0.05 *versus control group (MW); ^b^*p < 0.05 *versus KA group (MW). For the brainstem, descriptive data are presented as the mean ± SEM (n = 6 rats per group for each time point) and the significant difference was determined by parametric test, ANOVA test, followed by Tukey post hoc test with *p<0.05 *indicating significant difference. ^a^*p < 0.05 *versus control group; ^b^*p < 0.05 *versus KA group. ANOVA, one-way analysis of variance; GFAP, glial fibrillary acidic protein; IqR; interquartile range; KA, kainic acid; TH, tualang honey; TPM, topiramate; SEM, the standard error of the mean.

## Data Availability

The data used to support the findings of this study are available from the corresponding author upon request.
